# Genome-Wide Identification and Expression Analysis Reveal bZIP Transcription Factors Mediated Hormones That Functions during Early Somatic Embryogenesis in *Dimocarpus longan*

**DOI:** 10.3390/plants13050662

**Published:** 2024-02-28

**Authors:** Tingkai Zhai, Shuoxian Lan, Luzhen Xv, Xueying Zhang, Xiangwei Ma, Zhuoyun Li, Jie Gao, Yukun Chen, Zhongxiong Lai, Yuling Lin

**Affiliations:** 1 Institute of Horticultural Biotechnology, Fujian Agriculture and Forestry University, Fuzhou 350002, China; ztk1318347546@163.com (T.Z.); lanshuoxian2023@163.com (S.L.); 18950350912@163.com (L.X.); zxyying@163.com (X.Z.); 13348635486@163.com (X.M.); lizhuoyun970117@163.com (Z.L.); 17806853231@163.com (J.G.); cyk68@163.com (Y.C.); laizx01@163.com (Z.L.); 2 Key Laboratory of Genetics, Breeding and Multiple Utilization of Crops, Ministry of Education, Fujian Agriculture and Forestry University, Fuzhou 350002, China

**Keywords:** *Dimocarpus longan*, bZIP transcription factors family, bioinformatic evaluation, somatic embryogenesis, hormone

## Abstract

The basic leucine zip (bZIP) transcription factors (TFs) are a group of highly conserved gene families that play important roles in plant growth and resistance to adversity stress. However, studies on hormonal regulatory pathways and functional analysis during somatic embryogenesis (SE) in *Dimocarpus longan* is still unavailable. In this study, a total of 51 bZIP family members were systematically identified in the whole genome of longan, a comprehensive bioinformatics analysis of *DlbZIP* (*bZIP* family members of *D.* longan) was performed, and subcellular localization and profiles patterns after transiently transformed *DlbZIP60* were analyzed. The combined analysis of RNA-seq, ATAC-seq and ChIP-seq showed that four members have different H3K4me1 binding peaks in early SE and differentially expressed with increased chromatin accessibility. Comparative transcriptome analysis of bZIPs expression in early SE, different tissues and under 2,4-D treatment revealed that *DlbZIP* family might involved in growth and development during longan early SE. The qRT-PCR results implied that *DlbZIP* family were subjected to multiple hormonal responses and showed different degrees of up-regulated expression under indole-3-acetic acid (IAA), abscisic acid (ABA) and methyl jasmonate (MeJA) treatments, which indicated that they played an important role in the hormone synthesis pathways associated with the early SE of longan. Subcellular localization showed that DlbZIP60 was located in the nucleus, and the contents of endogenous IAA, MeJA and ABA were up-regulated in transiently *DlbZIP60* overexpressed cell lines. These results suggest that *DlbZIP60* may mediate hormones pathways that functions the development during early SE in longan.

## 1. Introduction

Basic leucine zip (bZIP) transcription factors (TFs) are a large and highly conserved family of genes in eukaryotes that affect and regulate the growth and development of plants [1], animals [2] and microorganisms [3]. In plants, bZIP family members are different in number due to evolutionary relationships, environmental conditions, and species. In 2002, Jakoby et al. [1] first classified 75 *Arabidopsis thaliana* bZIP family members into (A–I and S) 10 subfamilies using sequence similarity in the basic amino acid region as a differentiation criterion. Later, the *Arabidopsis thaliana* family members were updated to 78 by Droge-Laser et al. [4] on this basis (addition of bZIP76-bZIP79, exclusion of bZIP73) and expansion of the M, K, and J subfamilies. Currently, with reference to *Arabidopsis thaliana*’s criteria for the classification of bZIP family members, family members have been identified in *Oryza sativa* [5], potato (*Solanum tuberosum* L.) [6], cucumber (*Cucumis sativus* L.) [7], tomato (*Solanum lycopersicum* L.) [8], pepper (*Capsicum annuum* L.) [9], and grape (*Vitis vinifera*) [10]. It shows that bZIP transcription factors perform multiple functions and roles in different plant biological processes.

As a group of highly conserved gene families, the conserved structural domains of bZIP transcription factors are composed of 60~80 amino acid residues, including two conserved structural domains with different functions, the basic amino acid region and the leucine zip [11]. The basic amino acid region is located at the C-terminus and consists of 16−20 amino acid residues, which can bind to specific DNA sequences on the promoter [12]. The leucine zip region is located at the N-terminal end and consists of one or more repeating heptapeptides, with leucine at position seven of each repeating region, which is used to mediate homodimer and heterodimer formation of bZIP proteins [13]. The different conserved structural domains among the members of the bZIP family of transcription factors also contribute to the existence of different functions and mechanisms of action among the members. Overall, bZIP TFs mainly regulate the intensity of gene expression in response to various stresses in the external environment. In terms of growth and development, overexpression of the grape (*Vitis vinifera*) *VvbZIP36* gene can regulate the expression of ABA-related genes in *Arabidopsis thaliana*, enhance the antioxidant capacity of the plant, and improve the drought tolerance of *Arabidopsis thaliana* during seed germination [14]. Overexpression of wheat *TabZIP28 (bZIP28* of *Triticum aestivum)* gene could enhance seed germination rate of *Arabidopsis thaliana* under the environmental conditions of high temperature stress [15]. In addition, *Arabidopsis thaliana AtbZIP9* and *AtbZIP46* regulate the formation of floral meristematic tissue and vascular bundle development, respectively [16,17]. In terms of abiotic stress, silencing of the *SlbZIP1* gene can regulate the expression of ABA-related genes and enhance drought tolerance in tomato (*Solanum lycopersicum* L.) [18].The C/S1 bZIP network composed of MdbZIP2/39/80 (*Malus* × *domestica* Borkh.) can directly repress the expression of MdIPT5b, which can in turn enhance the drought tolerance of apple healing tissues [19]. Overexpression of bZIP60 enhances endoplasmic reticulum-related gene responses and improves heat tolerance in wheat [20], tomato (*Solanum lycopersicum* L.) [21], and maize [22]. Interaction of bZIP2 with bZIP3 in chrysanthemum [*Dendranthema gradifolium* (Ramat) Kitam] enhances antioxidant enzyme activities by regulating *DgPOD* expression, which in turn enhances chrysanthemum tolerance to low temperature [23]. In terms of biotic stress, salicylic acid (SA) content in *Arabidopsis thaliana* was increased by overexpression of the *Vitis vinifera VvbZIP60* gene, which resulted in up-regulated expression of *PR1* (*Pathogenesis related 1*) in the SA signalling pathway and improved disease resistance to powdery mildew [24]. Panax quinquefolius (*Panax quinquefolius* L.) *PqbZIP1* gene can enhance resistance to root rot by mediating various hormone signalling pathways such as jasmonic acid (JA), ABA and SA [25].

Longan (*Dimocarpus longan* Lour.) is a tropical/subtropical characteristic woody fruit tree native to China and Southeast Asia, belonging to the genus *D.* longan of the Sapindaceae *Dimocarpus* [26]. In China, production of *D.* longan has reached 1.85 million t in 2017 alone, with 340,000 ha under cultivation. In Thailand, *D.* longan was the second largest export crop after pineapple. In Vietnam, year-round production of *D.* longan can be achieved due to the climatic environment, with peak production in June and July. In 2017, *D.* longan was planted on 73,300 ha across Vietnam, producing about 550,000 t. The process of plant SE are highly similar to that of zygotic embryogenesis, and the SE system of *D.* longan established by Lai et al. [27] has a strong regenerative capacity and is highly synchronised, which is a good alternative system to study the development of *D.* longan SE. And the three stages of EC (embryogenic callus), IcpEC (incomplete compact pro-embryogenic cultures) and GE (globular embryos) in SE of *D.* longan were used as the main study. The publication of second-generation [28] and third-generation genomes [29] of *D.* longan has greatly facilitated the identification of gene families and functional studies. In recent years, gene families affecting SE in *D.* longan such as GLP [30], SAUR [31], XTH [32], DEAD-box [33], and Sm [34] have been identified, but genome-wide identification of bZIP TFs in *D.* longan has not been performed. Nowadays, it has been demonstrated that bZIP TFs respond to ABA-responsive elements (ABREs) of SE [35,36,37]. A recent study found that significant up-regulation of DEGs encoding AP2-EREBP, MYB, and bZIP TFs in *Oryza sativa* activates *CONSTITUTIVE PHOTOMORPHOGENIC 1* (*OsCOP1*), which affects the formation of embryonic structures in *Oryza sativa* seeds [38]. However, most of studies on bZIP TFs have focused on the mechanisms of response to adversity stress, but studies on their hormonal regulatory pathways and functions in the plant SE have not been reported.

In this study, we performed a comprehensive bioinformatics evaluation of bZIP TFs in the *D.* longan genome. A total of 51 family members of *D.* longan bZIP TFs were identified and analysed for their protein physicochemical properties, phylogenetic relationships, gene structures, covariance analysis, *cis*-acting elements and expression patterns under different treatments during early of SE in *D.* longan. RNA-seq, ATAC-seq and ChIP-seq were used to map alluvial diagrams of bZIP family members in *D.* longan and to analyse their chromatin accessibility. The qRT-PCR were used to analyse the response of *DlbZIP* family members during early SE and in response to different hormone treatments. And DlbZIP60 was screened for tertiary structure, protein interaction network, subcellular localization and transient transformation. These results will expand our understanding of DlbZIP TFs and their family members, and help to explore the mechanism of bZIP TFs during early SE in *D.* longan.

## 2. Results

### 2.1. Identification of DlbZIP Family Members and Analysis of Basic Physicochemical Properties

The 51 DlbZIP family members screened were named according to their similarity to *Arabidopsis thaliana* bZIP TFs. The smallest amino acid number was 89 aa, and the largest was 1545 aa. Molecular masses were concentrated between 11.38 kD and 177.87 kD. The distribution of isoelectric points ranged from 4.93 to 10.18. Among the instability coefficients, only DlbZIP23.1 and DlbZIP23.2 were <40, indicating that the proteins existed stably, while the others were unstable. 51 members of the DlbZIP family were hydrophilic and had no signal peptide. Most members are subcellularly localised to nucleus, except DlbZIP1 is localised to both nucleus and cytoplasm and DlbZIP17 is localised to endoplasmic reticulum (Table S1). 

### 2.2. Phylogenetic Analysis of DlbZIP Family Members

**Figure 1:**
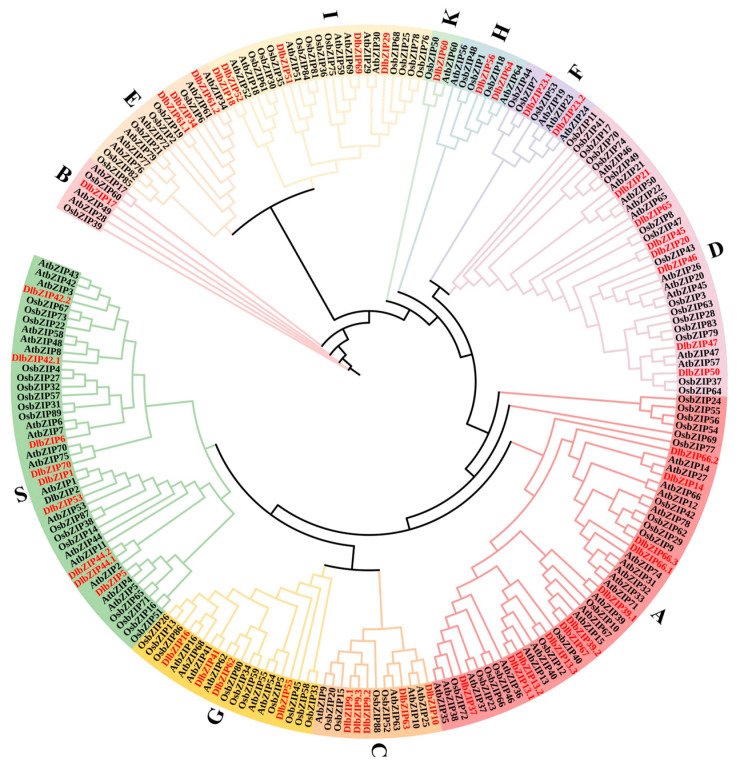
Phylogenetic analysis of bZIP family members in *D.* longan (Dl), *Arabidopsis thaliana* (At), and rice (Os, *Oryza sativa*).

In order to clarify the affinities of DlbZIP family members, we constructed a phylogenetic tree containing 218 bZIP family members, including *D.* longan (51 members), *Arabidopsis thaliana* (78 members) and *Oryza sativa* (89 members) (Figure 1). According to the bZIP family classification method [4], the phylogenetic tree can be divided into A, B, C, D, E, F, G, H, I, K, S eleven subfamilies. Among them, *D.* longan bZIP family members were most distributed in subfamilies A group, containing 11 family members. The next subfamilies was S, containing nine family members. It contains seven members in subfamilies D, five members in subfamilies C and I, four members in subfamilies G (DlbZIP16/41/55/62), three members in subfamilies E (DlbZIP34/61.1/61.2), two members in subfamilies F (DlbZIP23.1/23.2) and subfamilies H (DlbZIP56/64). Both subfamilies B (DlbZIP17) and subfamilies K (DlbZIP60) contain only one family member. Overall, members of the same bZIP family are heavily clustered in subfamily groups A, C, D, I, and S, which suggested that bZIP transcription factors are conserved. 

### 2.3. Conserved Motif and Gene Structure of DlbZIP Family Members

**Figure 2:**
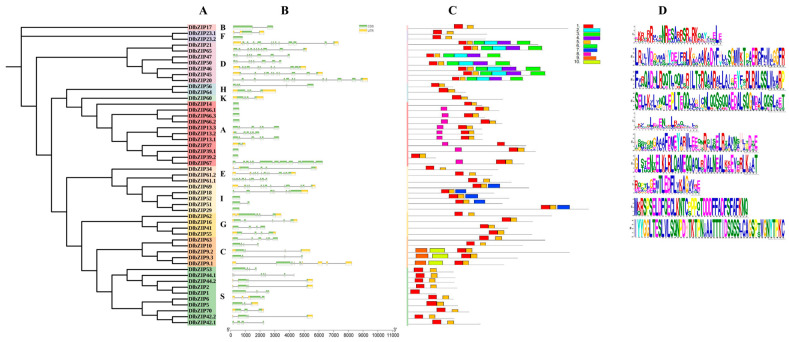
Phylogenetic tree, gene structures and conserved motifs of DlbZIP family members. (**A**) Phylogenetic analysis of bZIP family members in *D.* longan, (**B**) Gene structure location of bZIP family members in *D.* longan. Green boxes indicate exons; Yellow boxes indicate UTR; Black lines indicate introns. (**C**,**D**) The motif composition of DlbZIP proteins. The motifs, numbers 1–10, were displayed in different colored boxes.

To further understand the *D.* longan bZIP TFs, organization of intron/exon was detected via alignment of genomic DNA and open reading frame (ORF) sequences of *D.* longan bZIP family members (Figure 2). The gene structure results (Figure 2B) showed that *D.* longan bZIP family members have exon numbers ranging from 0–4 and intron numbers ranging from 0–12. The highest numbers of exons were found in DlbZIP9.1 (four numbers in total) and the highest numbers of introns were found in DlbZIP61.2 (12 numbers in total). In subfamily, some members showed similarity in gene structure. DlbZIP25/46/65 in subfamily D all contain two exons and 10 introns, and DlbZIP47/50 all contain seven introns. DlbZIP13.1/13.2/13.3 in subfamily A all contain five introns. Subfamily G DlbZIP41/55 both contain three introns, and DlbZIP16/62 both contain four introns. Most of S subfamily members contain three introns. Overall, there were large variations among DlbZIP family members, which suggested a complex mechanism in their evolution.

Conservative motif results (Figure 2C) showed that 13 subfamilies shared motif 1, and all but DlbIP1 shared motif 5. Motifs 2, 3, 4, and 6 were specific to subfamily D. Motif 7 was specific to subfamily I. All subfamily A, except DlbIP39.2, shared motif 8. Motif 9 was specific to DlbIP9.1, DlbIP9.2, and DlbIP9.3. Overall: The gene structures of the bZIP family members differ greatly from each other, which suggests a complex mechanism in their evolution. Members of the same subfamily contain motifs with some similarity, and it is speculated that each subfamily may have different functional roles.

### 2.4. Chromosomal Localization, Covariance Analysis and Cis-Acting Element of DlbZIP Family Members

**Figure 3:**
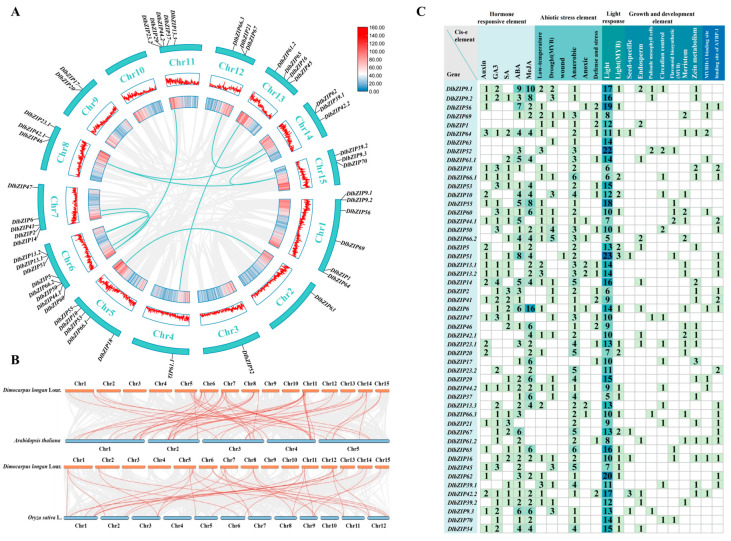
Chromosomal localisation, covariance analysis and *cis*-acting element analysis of DlbZIP family members. (**A**) Chromosome location and collinearity analysis of *DlbZIP* family members. Gene pairs located in the segmental duplicated chromosomal regions were linked using different lines. (**B**) Synteny analysis of *bZIP* between *D.* longan and two model plants(*Arabidopsis thaliana* and *Oryza sativa*). Gray lines in the background indicate the collinear blocks within *D.* longan and other plant genomes, while the red lines highlight the syntenic *bZIP* gene pairs. (**C**) *Cis*-acting elements of *DlbZIP* family members.

In order to further understand the gene-spotting information, potential genetic mechanisms of evolution and functional roles of *DlbZIP* family members on chromosomes, we performed chromosomal localization, covariance analysis and *cis*-acting element analysis of *DlbZIP* family members. The results of chromosomal localization showed that there were 50 (except *DlbZIP34*) *DlbZIP* family members unevenly distributed on 14 chromosomes (Figure 3A), with the most *DlbZIP* family members distributed on chromosome 6 and not found in chromosome 10. Gene tandem duplications were present on Chr 1, 6, 8, 9, 11, 12, and 14. A total of 10 co-linear pairs were found distributed on 11 chromosomes (except Chr 1, Chr 3, Chr 9, and Chr 10). Overall, tandem duplication and segmental duplication events existed for *DlbZIP* in *D.* longan, and we hypothesised that most of the *DlbZIP* family members might have arisen from gene duplication and driven the evolution of *DlbZIP* family members. Meanwhile, we constructed a homology analysis network between *D.* longan and two model plants (*Arabidopsis thaliana* and *Oryza sativa*) (Figure 3B), which was consistent with the results derived from the phylogenetic tree that *D.* longan has high homology with dicotyledonous plants (*Arabidopsis thaliana*).

To clarify the potential functions of *DlbZIP* family members in response to various responses and to explore their functions, we predicted *cis*-acting elements 2000 bp upstream of CDS (Figure 3C). The results showed that all *DlbZIP* family members contained hormone-related response elements except *DlbZIP1* and *DlbZIP63*, and the percentage of response elements was higher in abscisic acid (ABA) and methyl jasmonate (MeJA). In terms of stress response, there were 26 *DlbZIP* family members responding to low temperature stress, 24 *DlbZIP* family members responding to drought stress, 48 *DlbZIP* family members responding to injury, eight *DlbZIP* family members responding to hypoxia stress, and 18 DlbZIP family members responding to defence and stress. All *DlbZIP* family members contained light-responsive elements, with fewer response elements related to growth and development. These results indicated that *DlbZIP* may be involved in a wide range of stress responses and hormone regulation and play a wide range of roles.

### 2.5. DlbZIP Family Members Respond to Early SE and Different Developmental Organs in D. longan

To further understand the changes in chromatin accessibility of *DlbZIP* family members at early SE, firstly, we analysed the ATAC-seq data and found that, except for *DlbZIP9.1*/*9.2*/*13.1*/*13.2*/*34*/*39.2*/*46*/*52*/*60*/*67*, other *DlbZIP* family members at early SE chromatin accessibility was open. Then, we performed ChIP-seq analysis of histone H3K4me1 and found that most of *DlbZIP* family members that were differentially expressed in early SE were not associated with H3K4me1 signalling enrichment. Finally, combined analysis with RNA-seq, ATAC-seq and ChIP-seq revealed that *DlbZIP6*, *DlbZIP42.1*, *DlbZIP50* and *DlbZIP53* had distinct H3K4me1 binding peaks and were differentially expressed with increased chromatin accessibility during early SE (Supplementary Figure S1).

**Figure 4:**
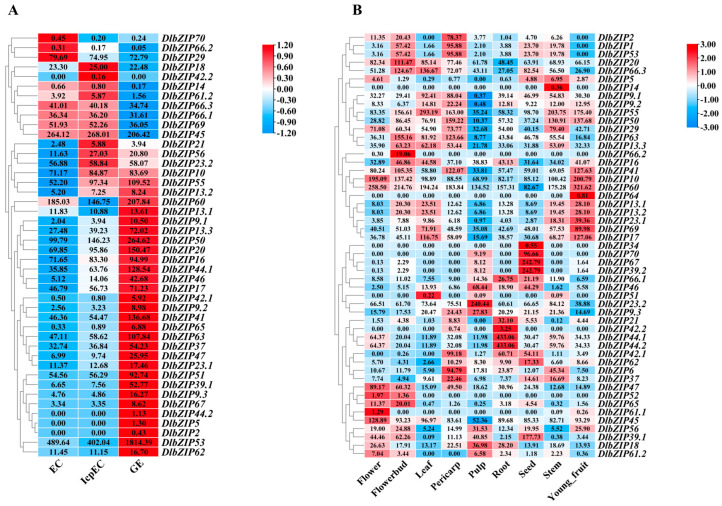

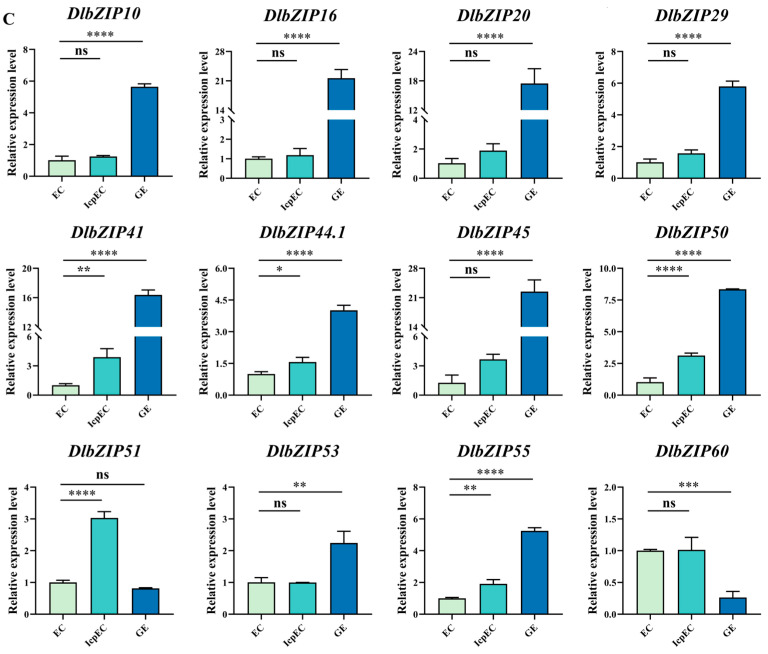
Expression profile during early SE and different developmental organs. (**A**,**B**) The remaining clusters based on expression analyses of *DlbZIP* using previously published transcriptome data from *D.* longan early SE and different developmental organs. (**C**) Relative expression of *DlbZIP* family members during early SE in *D.* longan. Figure “*” is *p* < 0.05, “**” is *p* < 0.01, “***” is *p* < 0.001, “****” is *p* < 0.0001, “ns” is not significantly different.

Analysis of FPKMs expressed by *DlbZIP* family members during early SE (Figure 4A) showed that 44 of *DlbZIP* family members showed different levels of expression, of which five had very low levels of expression (FPKMs < 1 at all three stages). And among other 39 members, three were preferentially expressed in EC, eight were preferentially expressed in IcpEC, and 28 were preferentially expressed in GE. Taken together, members of *DlbZIP* family are involved in the formation of GE stage and promote early SE in *D.* longan. FPKMs in nine different developmental organs of *D.* longan were screened and analysed from the transcriptome database (Figure 4B), and the results showed that 51 *DlbZIP* family members were expressed in different degrees in nine different developmental organs of *D.* longan. Among them, *DlbZIP10*, *DlbZIP20*, *DlbZIP45*, and *DlbZIP60* showed high expression in each developmental organ, which are presumed to be widely involved in various stages of *D.* longan growth and development. Among them, 25 family members were up-regulated in flower bud, 23 family members in stem, and only 11 family members in pulp. *DlbZIP14* was expressed only in stem, *DlbZIP64* only in young fruit, and *DlbZIP34* only in seed. In summary, *DlbZIP* family members respond to the nine developmental organs of *D.* longan in different degrees and participate in different processes of *D.* longan growth and development.

To further illustrate the results of the transcriptomic data, we screened a total of 12 *DlbZIP* family members that were highly expressed in two transcriptomes associated with *D.* longan growth and development. The qRT-PCR was used to verify the expression of the 12 *DlbZIPs* during early *D.* longan SE (Figure 4C). The results showed that a total of 10 family members (DlbZIP*10*/*16*/*20*/*29*/*41*/*44.1*/*45*/*50*/*53*/*55*) showed a gradual upward trend from EC to GE and peaked in GE. *DlbZIP51* was up-regulated and expressed in IcpEC, *DlbZIP60* was significantly down-regulated in GE. The qRT-PCR results of the three stages during early SE of *D.* longan showed some difference with the above heatmap, which led to the speculation that *DlbZIP* family members have a certain spatio-temporal expression specificity. These results indicated that 10 family members of the 12 *DlbZIPs* screened may play important roles in the development of GE, *DlbZIP51* and *DlbZIP60* may have a significant role in maintaining morphogenesis during early SE in *D.* longan.

### 2.6. DlbZIP Family Members Are Involved in Multiple Hormone Transduction Pathways

**Figure 5:**
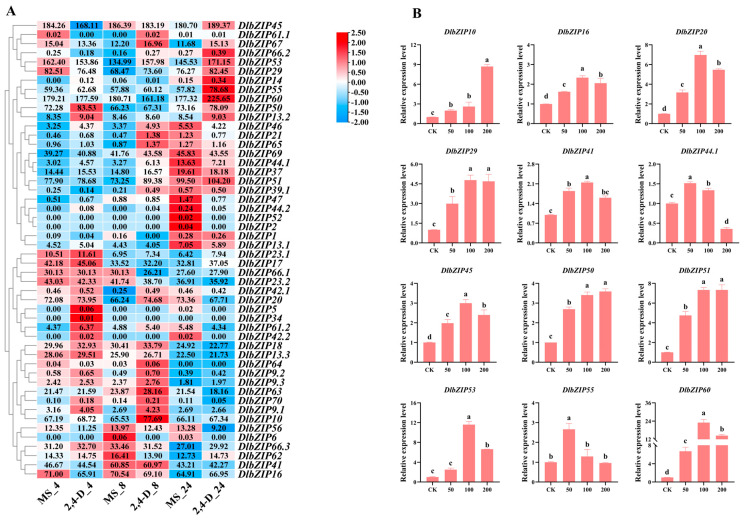

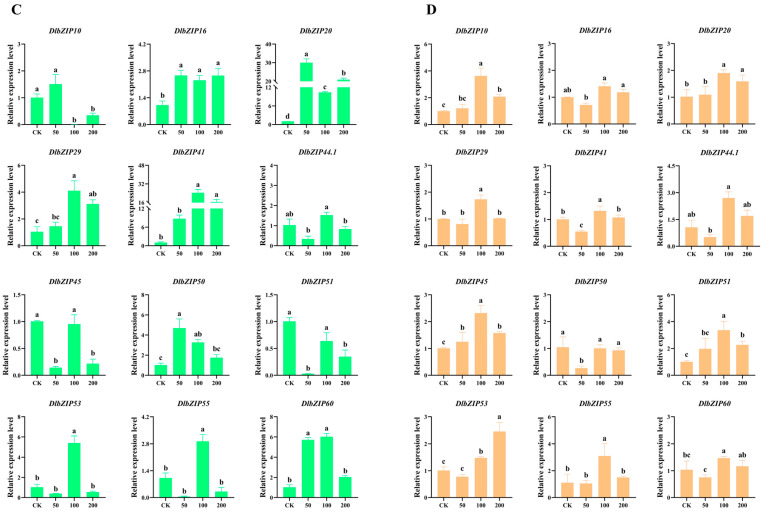
Expression analysis of *D.* longan EC under different hormone treatments. (**A**) Expression of DlbZIP in EC under 2,4-D treatment. From left to right respectively represent 4 h, 8 h and 24 h treatment of *D.* longan EC using MS media and 2,4-D media. (**B**) Expression of *DlbZIP* family members in *D.* longan EC at different concentrations of IAA. (**C**) Expression of *DlbZIP* family members in *D.* longan EC at different concentrations of ABA. (**D**) Expression of *DlbZIP* family members in *D.* longan EC at different concentrations of MeJA. Different letters indicate statistically significant differences (*p* ≤ 0.05).

Hormones are major factors affecting SE development, and promoter cis-acting element analyses revealed that multiple hormone-responsive elements exist in *DlbZIP* family members. Combined with the results of the above experiments, the expression profiles of *DlbZIP* family members were analysed at 2,4-D hormone treatment (Figure 5A). The expression results showed that most *DlbZIP* family members were expressed higher in the 2,4-D hormone-treated group than that in the control group (MS) at the same treatment time, and were highly expressed at 8 h versus 24 h of 2,4-D hormone treatment, and collectively belonged to the fact that most of the *DlbZIP* family members were responsive to auxin hormone.

Based on the *cis*-acting element analysis and expression analysis of *D.* longan EC under 2,4-D hormone treatments of *DlbZIP* family members, *D.* longan EC was treated with different concentrations (0 μM, 50 μM, 100 μM, and 200 μM) of IAA, ABA, and MeJA. And the expression levels of the 12 *DlbZIP* family members were detected by qRT-PCR under each hormone treatment. The results of the expression levels of the 12 *DlbZIPs* under IAA treatment (Figure 5B) showed that, except for *DlbZIP44.1*, a trend of up-regulation of the expression of the remaining 11 *DlbZIP* were observed under IAA treatment. Two *DlbZIP* (*DlbZIP44.1*/*55*) had the highest expression under IAA treatment at 50 μM, seven *DlbZIP* (*DlbZIP16*/*20*/*29*/*41*/*45*/*53*/*60*) had the highest expression under 100 μM IAA treatment, and three *DlbZIP* (*DlbZIP10*/*50*/*51*) had the highest expression under 200 μM IAA treatment.

Analysis of the expression levels of 12 *DlbZIPs* under ABA treatment (Figure 5C) revealed that 12 *DlbZIPs* showed different levels of expression under different concentrations of ABA, among which *DlbZIP16*/*20*/*29*/*41*/*50*/*60* showed up-regulation of expression under all three concentrations of ABA. The expression of *DlbZIP44.1*/*53*/*55* was only up-regulated under 100 μM ABA treatment, and was lower than that of the control group under 50 μM and 200 μM ABA treatments. *DlbZIP45*/*51* showed a trend of down-regulated expression under all three ABA concentration treatments, and *DlbZIP10* expression was slightly up-regulated under 50 μM ABA treatment, but it was not significantly different from the control. The results showed that, different *DlbZIPs* play different functions during early *D.* longan SE, among which *DlbZIP16*/*20*/*29*/*41*/*50*/*60* TFs may be involved in the ABA signalling pathway during early *D.* longan SE.

Analysis of the expression levels of 12 *DlbZIPs* under MeJA treatment (Figure 5D) revealed that most of *DlbZIP* showed up-regulated expression trends under 100 μM and 200 μM MeJA treatments, with the highest expression under 100 μM MeJA treatment (only *DlbZIP53* showed the highest expression under 200 μM MeJA treatment). While, under 50 μM MeJA treatment, *DlbZIP16*/*20*/*29*/*41*/*44.1*/*50*/*53*/*55*/*60* were down-regulated. 

### 2.7. Tertiary Structure and Protein Interaction Networks of the DlbZIP60

**Figure 6:**
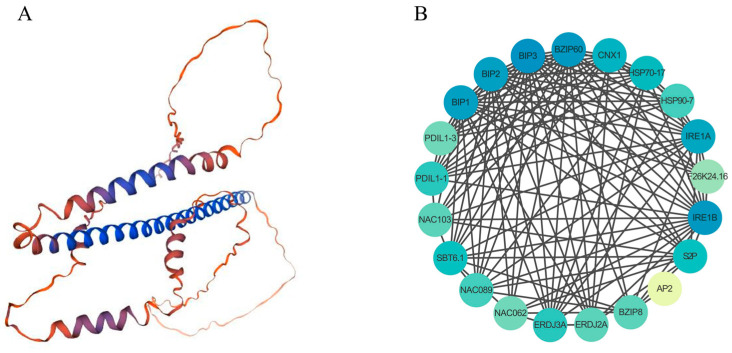
Tertiary structure and protein interaction network of DlbZIP60. (**A**) Tertiary structure of DlbZIP60. (**B**) Protein interaction network of DlbZIP60. Network nodes represent proteins.

Jakoby et al. (2002) performed the first genome-wide identification of *Arabidopsis thaliana* bZIP TFs, in which AtbZIP60 was not distinguished into any of the subgroups due to its structural specificity. Droge-Laser et al. (2018) updated the number of family members and subgroups of *Arabidopsis thaliana* bZIP TFs separately, AtbZIP60 was defined separately in the K subfamily. It is hypothesised that AtbZIP60 may have a unique function. Consistent with the phylogenetic tree results in this paper, it was found that DlbZIP60 was co-distinguished from AtbZIP60 and OsbZIP50 in the K subfamily. Combined with the results of multiple transcriptomics and qRT-PCR analyses, it was hypothesised that DlbZIP60 likewise has certain unique structures and functions. Thus, we performed tertiary structure for DlbZIP60 (Figure 6A), and found that the structure of DlbZIP60 transcription factor existed 48.34% of irregular coiling followed by α-folding, accounting for 43.71%; extended strand accounted for 6.29%, and β-folding accounted for 1.66%. From the protein interaction network (Figure 6B), it can be seen that the DlbZIP60 transcription factor has strong protein interactions with a variety of transcription factors and genes in *D.* longan. Among them, there was a close interprotein interaction with the heat stress proteins HSP70-17 and HSP90-7 and their molecular chaperones BIP1, BIP2 and BIP3 proteins, and there was an interaction with key plant transcription factors such as NAC and AP2, implying that DlbZIP60 transcription factors played an important role in the growth and development of *D.* longan.

### 2.8. DlbZIP60 Is Located in the Nucleus and Affects the Content of Multiple Endogenous Hormones in D. longan EC

**Figure 7:**
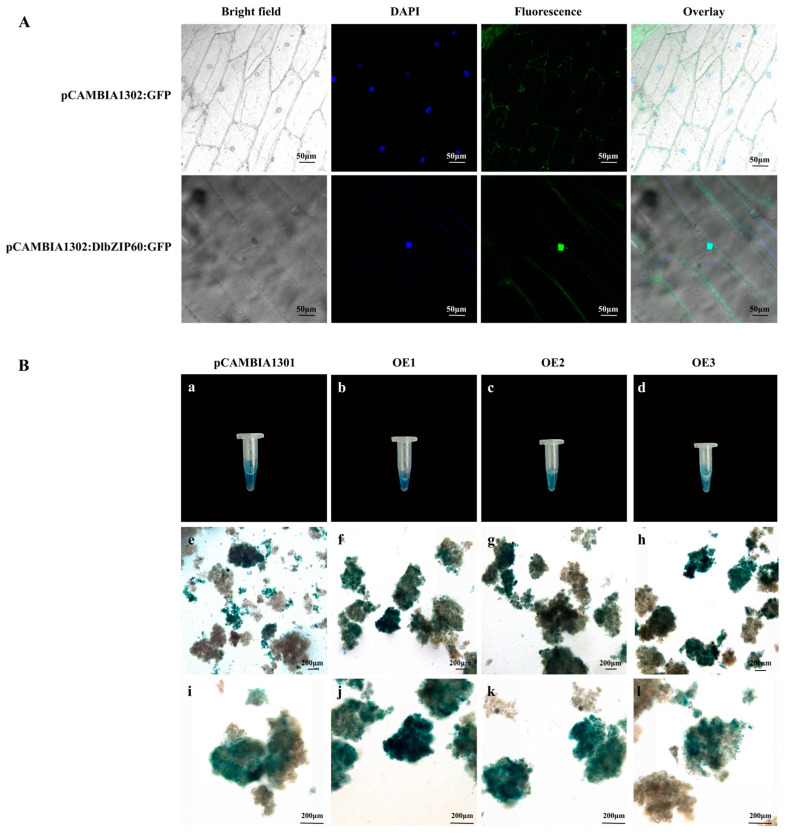
Subcellular localization and transient transformation of DlbZIP60. (**A**) Subcellular localization of the DlbZIP60-GFP. (**B**) GUS staining and microscopic observations of pCAMBIA1301 and *DlbIP60* after transient transformation. The (**e**–**h)** are fluorescence microscopic observations of pCAMBIA1301 and *DlbIP60* cell lines at 10× magnification. The (**i**–**l**) are fluorescence microscopic observations of pCAMBIA1301 and *DlbIP60* cell lines at 20× magnification.

The DlbZIP60 protein was predicted to be located in the nucleus based on analysis of the physicochemical properties of DlbZIP family member proteins. To verify the accuracy of the prediction results, we used onion for subcellular localization. The experimental results revealed that after performing onion infiltration, the fluorescence signals of pCAMBIA1302:GFP (Figure 7A) were distributed in the cell wall and nucleus, and the fluorescence signals of pCAMBIA1302:DlbZIP60:GFP were enhanced in the nucleus, and the overlap of DAPI and fluorescence field in the nucleus, which verified that the localization of DlbZIP60 proteins was in accordance with the predicted results. 

The pCAMBIA1301 and pCAMBIA1301:DlbZIP60:GUS construct were transformed into *Agrobacterium tumefaciens* (EHA105). After co-culturing in MS solid medium for 3 d, the transiently transformed *D.* longan EC were stained with GUS. The results revealed that all pCAMBIA1301 and DlbZIP60 overexpression cell lines (OE1-OE3, three cell lines in total) were found to turn blue. And positive cell lines that had turned blue were clearly detected with microscopy (Figure 7(Be–Bl)). 

**Figure 8:**
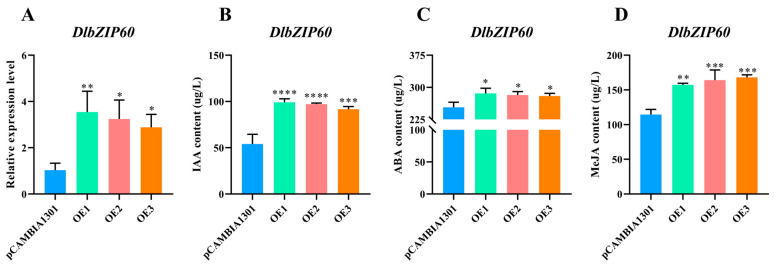
Expression analysis and endogenous hormone changes after transient transformation of *D.* longan EC by *DlbZIP60*. Figure “*” is *p* < 0.05, “**” is *p* < 0.01, “***” is *p* < 0.001, “****” is *p* < 0.0001. (**A**) Relative expression level of pCAMBIA1301 and *DlbZIP60* after transient transformation. (**B**–**D**) IAA, ABA, MeJA content of pCAMBIA1301 and *DlbZIP60* after transient transformation.

Expression analyses and hormone content measurements of the positive cell lines after transiently transformed showed that the expression levels of *DlbZIP60* were significantly elevated in qRT-PCR validation compared to pCAMBIA1301 (Figure 8A), and endogenous IAA, ABA, and MeJA contents appeared to be increased in different levels (Figure 8B–D). The relative expression level, IAA content and ABA content of OE1 were up-regulated higher than other two overexpression-positive cell lines, presumably due to the highest infiltration efficiency of this cell line. Based on the above results, we speculate that *DlbZIP60* is involved in multiple hormone signal transduction pathways in *D.* longan, influencing the SE genesis process by regulating IAA content, and responding to various types of stresses in the external environment by regulating ABA and MeJA content.

## 3. Discussion

### 3.1. DlbZIP Family Members Are Evolutionarily Conserved and Functionally Diverse

As an important transcription factor affecting plant growth and adversity stress, bZIP transcription factors are important for enhancing responses to various types of stresses in the external environment by participating in ABA signal transduction and expression of related genes [14,21,25]. Currently, with reference to *Arabidopsis thaliana*’s criteria for the classification of bZIP family members, 89 family members (divided into A–J with 10 subfamilies) have been identified in *Oryza sativa* [5], 56 family members (divided into A–J with 10 subfamilies) in potato (*Solanum tuberosum* L.) [6], 64 family members (divided into 8 subfamilies Ia–c and II–VI with 8 subfamilies) in cucumber (*Cucumis sativus* L.) [7], 69 family members (divided into A–I with 9 subfamilies) in tomato (*Solanum lycopersicum* L.) [8], 54 family members (divided into A–I and S with 10 subfamilies) in pepper (*Capsicum annuum* L.) [9], and 55 family members (divided into A–J with 10 subfamilies) in grape (*Vitis vinifera*) [10]. In this study, we screened 51 unduplicated bZIP family members from *D.* longan, which are much lower in number than *Arabidopsis thaliana* [4] and *Oryza sativa* [5]. And it is similar to potato (*Solanum tuberosum* L.) [6], pepper (*Capsicum annuum* L.) [9] and grape (*Vitis vinifera*) [10]. The difference in the number of bZIP family members in *D.* longan and other species may be caused by the presence of fewer tandem versus segmental repeat events in *D.* longan. In past studies, a large number of tandem repeats were found in both cucumber (*Cucumis sativus* L.) [7] and tomato (*Solanum lycopersicum* L.) [8], favouring the occurrence of different degrees of expansion in the number of family members. In summary, the number of bZIP family members in *D.* longan is less than that in other plants, probably because some of the DlbZIP family members are generated by gene duplications and drive the evolution of DlbZIP family members.

Based on the comprehensive phylogenetic tree containing *D.* longan, *Arabidopsis thaliana*, and *Oryza sativa* bZIP, we classified *D.* longan bZIP into 11 subfamilies: A–I, K, and S. The results showed that although different *D.* longan bZIP differed in physicochemical properties, they were members of the same subfamily and contained motifs with certain similarities, which suggested that each subfamily might have different functional roles. Combined with the prediction of *cis*-acting elements 2000 bp upstream of CDS, it was found that *DlbZIP* may be involved in various stress responses (low temperature, drought, injury, hypoxia) and hormone regulation (IAA, ABA, and MeJA). This also explains that *DlbZIP* play important roles in growth and resistance to biotic/abiotic stresses in *D.* longan through different *cis*-acting elements.

### 3.2. DlbZIP Family Members Involved in Early D. longan SE and Related Hormone Synthesis Pathways

As a TF which affects plant growth and development, it has been shown that bZIP TFs can make ABREs of SE responsive [37]. *Arabidopsis thaliana* bZIP60 interacts with bZIP17 to promote seed germination [38]. Up-regulation of differentially expressed genes of bZIP TFs promotes the formation of embryonic structures in *Oryza sativa* [39]. In our study, we found that the transcriptome data showed a large number of *DlbZIP* were highly expressed in GE and showed a gradual up-regulated expression trend during early *D.* longan SE (EC-IcpEC-GE), which was also verified in qRT-PCR validation of the three stages of SE. The screening of nine *D.* longan developmental organ FPKMs from the transcriptome database revealed that members of *DlbZIP* family responded to the nine developmental organs of *D.* longan to varying degrees, and participated in different processes of *D.* longan during growth and development. 

It has been well documented that bZIP TFs control a variety of important signalling molecules in the plant hormone transduction pathway and interact with genes as important players in plant resistance to adversity. For example, members of the bZIP transcription factor A subfamily can bind directly to abscisic acid-responsive cis-elements (ABRE) and participate in the abscisic acid pathway [40]. AcTGA01, AcTGA06, and AcTGA07 in kiwifruit (*Actinidia*) can respond to different levels of the hormones and enhance resistance to ulcer disease [41]. In our study, we found that *DlbZIP* may be involved in multiple hormone regulation (IAA, ABA, MeJA) processes. Meanwhile, the expression profiles of 2,4-D hormone treatment showed that most *DlbZIP* family members showed up-regulated expression under 2,4-D hormone treatment compared with MS treatment, and the expression increased with the treatment time. It suggests that members of *DlbZIP* family are responsive to growth hormone during early *D.* longan SE. To further study the effect of hormones on SE in *D.* longan, we treated *D.* longan EC with different concentrations of IAA, ABA, and MeJA (all four concentrations of 0 μM, 50 μM, 100 μM, and 200 μM) and verified them by qRT-PCR, which showed that 12 *DlbZIP* were up-regulated under the treatment of different concentrations of IAA, and most of *DlbZIP* were up-regulated under the treatment of ABA and MeJA. In summary, *DlbZIP* may act during early SE in *D.* longan and are closely related to the IAA synthesis pathway.

### 3.3. Overexpression of DlbZIP60 May Be Involved in the Regulation of Embryogenesis in SE of D. longan

In two classifications of *Arabidopsis thaliana* bZIP family members, AtbZIP60 was always in a relatively independent position. It is speculated that bZIP60 may have a unique function. A previous study showed that under adversity stress, the conserved double stem-loop structure of the mRNA in bZIP60 can be localised to the endoplasmic reticulum by IRE1 splicing, which in turn encodes the bZIP60 protein without transmembrane domains, and is transferred to the nucleus to initiate the transcription of downstream genes [42]. In this study, subcellular localization of DlbZIP60 containing a GFP tag was performed on onion after infestation, and the results showed that it was localised to the nucleus. Previous studies have found that Toothbrush (*Aquilaria sinensis*) AsbZIP14-GFP or AsbZIP41-GFP [43], Bletilla (*Bletilla striata*) BsbZIP13-GFP [44], Tomato (*Solanum lycopersicum* L.) SlbZIP06 -GFP, SlbZIP12-GFP, SlbZIP16-GFP, SlbZIP32-GFP and SlbZIP46-GFP [8] were all located in the nucleus. This observation indicated that DlbZIP60, which may use the nucleus as the beginning of transcription, involves in multiple hormonal regulatory pathways in *D.* longan and affecting SE.

The bZIP TFs are one of the four major families of stress tolerance-associated TFs (bZIP, WRKY, MYB, and NAC) in plants, which are involved in a variety of growth and developmental processes. It has been found that overexpression of bZIP60 enhances endoplasmic reticulum-related gene responses and improves heat tolerance in wheat [20], tomato (*Solanum lycopersicum* L.) [6], and maize [22]. In this study, to verify the effects produced by overexpression of *DlbZIP60* in *D.* longan SE, transient transformation of *D.* longan EC was performed with *DlbZIP60* containing a GUS tag, and it was found that the endogenous IAA, ABA and MeJA contents of *D.* longan SE showed different levels of elevation after overexpression of *DlbZIP60*. Combined with the above qRT-PCR results after treatment with different concentrations of IAA, ABA and MeJA, we hypothesised that *DlbZIP60* could be involved in a variety of hormonal pathways during early *D.* longan SE development, and that changes in the content of endogenous hormones were positively correlated with the expression of *DlbZIP60*. Specifically, overexpression of *DlbZIP60* promotes the development of SE in *D.* longan by participating in the IAA synthesis pathway, and improves the response of *D.* longan SE to various kinds of stress environments by participating in the ABA and MeJA synthesis pathways, thus enhancing the resistance ability in adversity.

## 4. Materials and Methods

### 4.1. Plant Materials

Taking the ‘Honghezi (HHZ)’ *D.* longan SE as the primary material, including three stages of embryogenic callus (EC), incomplete compact pro-embryogenic culture (IcpEC) and globular embryo (GE), which were obtained in accordance with the method of the laboratory’s previous study [45]. Selected 0.2 g EC were treated with IAA, ABA and MeJA in MS medium at concentrations of 50 μM, 100 μM and 200 μM, which were incubated in the dark environment at 25 °C with 120 r·min^−1^ for 24 h. EC in MS medium without IAA, ABA and MeJA treatments were used as the control, and other conditions were unchanged. Three biological replicates were performed for each treatment, and the samples were collected and frozen in liquid nitrogen and stored in a −80 °C refrigerator. The onions (*Allium cepa* L.) used for subcellular localization were purchased from the market around the laboratory. The transiently transformed pCAMBIA1301 and pCAMBIA1301:DlbZIP60:GUS (OE1–OE3) cell lines were collected according to 0.1 g each. After freezing in liquid nitrogen, they were placed in a −80 °C refrigerator for backup. Used for qRT-PCR assay and endogenous hormone content determination. 

### 4.2. Identification and Protein Physicochemical Properties of D. longan bZIP Family Members

The amino acid sequences of *Arabidopsis thaliana* bZIP family members were obtained from The *Arabidopsis* Information Resource [46] (TAIR; https://www.arabidopsis.org/) (Accessed on 27 August 2022). Firstly, the *Arabidopsis thaliana* bZIP amino acid sequence was used as a search sequence and a probe to download the ‘HHZ’ *D.* longan third-generation genome from the National Center for Biotechnology Information (NCBI) Sequence Read Archive (SRA) database (SRR17675476) [29]. Searching for possible DlbZIP sequences was performed using the single Blast function of the TBtools software and further screened by two-way Blast at NCBI [47]. Second, the bZIP conserved structural domains of the screened members were reconfirmed using the HMMER online software [48] (https://www.ebi.ac.uk/Tools/hmmer/search/phmmer) (Accessed on 29 August 2022), and it was initially determined that the DlbZIP family contained a total of 51 members. Finally, the preliminarily identified members were compared with the ‘HHZ’ *D.* longan third-generation genome to search for any omissions and finally confirmed the existence of a totally 51 DlbZIP family members that are named in reference to the *Arabidopsis thaliana* nomenclature for the DlbZIP transcription factor family [4].

The number of amino acids (aa), molecular weight (MW) and isoelectric point (pl) of DlbZIP family proteins were analysed using the online software ExPASy [49] (https://web.expasy.org/protparam/) (Accessed on 4 September 2022); the signal peptides and subcellular localization of the DlbZIP family proteins were predicted by using the online software SignalP 4.1 respectively (http://www.cbs.dtu.dk/services/SignalP/) (Accessed on 4 September 2022) and WoLF PSORT [50] (https://wolfpsort.hgc.jp/) (Accessed on 23 November 2022).

### 4.3. Phylogenetic Tree, Conserved Motif and Gene Structure of DlbZIP Family Members

Amino acid sequences of *Oryza sativa* bZIP family members were obtained from Ensembl Plants [51] (http://plants.ensembl.org/) (Accessed on 5 October 2022). The phylogenetic tree was constructed by analysing *Arabidopsis thaliana*, *Oryza sativa* and *D.* longan using the maximum likelihood (ML) algorithm with TBtools software (TBtools V1.098.) (Accessed on 17 February 2023), and the phylogenetic tree was embellished by the online interactive software iTOL [52] (https://itol.embl.de/) (Accessed on 19 February 2023). 

*D.* longan single-species phylogenetic trees were constructed in the same way as described above. Using the online website MEME [53] (http://meme-suite.org/) (Accessed on 18 May 2023), the query base was set to 10 and other parameters were kept as default to search for possible motifs of DlbZIP members, and the analysed motifs were downloaded with The logo map. The gene structures of DlbZIP family members were analysed visually using TBtools based on the *D.* longan genome gff annotation file.

### 4.4. Chromosomal Localization, Covariance Analysis between Multiple Species and Cis-Acting Elements of DlbZIP Family Members

Chromosomal localization of DlbZIP family members was visualised using TBtools software based on the *D.* longan genome gff annotation file. Covalent gene pairs were obtained and visualised in TBtools software. Dual system Plotter software (https://github.com/CJ-Chen/TBtools) (Accessed on 24 May 2023) was used to construct covariance analysis maps for *D.* longan and *Arabidopsis thaliana*, as well as *D.* longan and *Oryza sativa*, respectively [54]. TBtools was used to extract parameters 2000 bp upstream of the *DlbZIP* family members CDS (Coding Sequence), and the results were submitted to the online website PlantCARE (http://bioinformatics.psb.ugent.be/webtools/plantcare/html/) (Accessed on 25 May 2023) for *cis*-acting element prediction and finally visualised the results in TBtools.

### 4.5. Analysis of Specific Expression of DlbZIP Family Members

The ATAC-seq (SRR18028214, SRR18028213, and SRR18028212) and the ChIP-seq (H3K4me1) (SRR18035255, SRR18035254, and SRR18035253) date during early *D.* longan SE (EC, ICpEC, and GE) were downloaded from the NCBI database. To download transcriptome during early *D.* longan SE of DlbZIP family members including EC (SRR21979789, SRR21979788 and SRR21979787), IcpEC (SRR21979786, SRR21979785 and SRR21979784) and GE (SRR21979783, SRR21979782 and SRR21979781). To download transcriptome data of early *D.* longan SE treated with 2,4-D (SRR21980424, SRR21980423, SRR21980414, SRR21980413, SRR21980412, SRR21980411, SRR21980410, SRR21980409, SRR21980408, SRR21980407, SRR21980422, SRR21980421, SRR21980420, SRR21980419, SRR21980418, SRR21980417, SRR21980416, SRR21980415). To download transcriptome data of different developing organs of *D.* longan (NCBI BioProject number: PRJNA326792). The FPKMs of *DlbZIP* family members in each data were extracted and analysed for expression with HeatMap by using TBtools.

### 4.6. The qRT-PCR Analysis of DlbZIP Family Members during Early SE, under Different Hormone Treatments and Transient Transformation

RNA was extracted using the TransZol Up Kit (All Style Gold, Beijing, China) according to the manufacturer’s instructions. The cDNA was synthesised with Revertaid Master Mix (Thermo Fisher Scientific, Shanghai China) and qRT-PCR was performed on the Roche Light Cycler 96 instrument by using the 10-fold diluted cDNA as amplification template. The data were calculated according to the 2^−ΔΔCt^ method based on UBIQUITIN (UBQ) as the internal reference. The data were analysed and processed with Duncan’s method in one-way ANOVA by SPSS 20 software. Visual plots were carried out using Prism 8.0.2 software. (Figure “*” is *p* < 0.05, “**” is *p* < 0.01, “***” is *p* < 0.001). The qRT-PCR primers for *DlbZIP* family members were designed with the DNAMAN 6.0 software (Table S2.).

### 4.7. Tertiary Structure and Protein Interaction Networks of DlbZIP Family Members

The DlbZIP60 tertiary structure was predicted through the SWISS-MODEL website [55] (https://swissmodel.expasy.org/) (Accessed on 1 July 2023). Protein interaction network prediction was performed using the online web site STRING [56] (https://cn.string-db.org/) (Accessed on 3 July 2023) and the results were embellished by Cytoscape software (Cytoscape 3.10) (Accessed on 3 July 2023) [57]. 

### 4.8. Subcellular Localization Analysis

The full-length coding sequence of Dlo013167 (DlbZIP60) without the stop codon was amplified with primers and cloned into the pCAMBIA1302-35S-GFP vector (Table S3.). The bacteriophage containing the recombinant plasmid was activated and cells were collected by centrifugation at 5000 r/min for 10 min. Resuspension of the cells was carried out by using infiltration solution containing 500 µmol·L^−1^ of 2-(N-morpholino)ethanesulfonic acid monohydrate (MES Free Acid, Monohydrate), 100µmol·L^−1^ of acetosyringone (AS) and 10 µmol·L^−1^ of MgCl_2_. The cells were resuspended and adjusted to OD_600_ between 0.6 to 0.9. The prepared infiltration solution was injected into the inner epidermis of onion and incubated in the dark at 25 °C for 3 d. At the end of the incubation, the cells were placed under the laser confocal microscope (Olympus FV 1000) (Manufacturer: Olympus Corporation) for observation and photographed. 

### 4.9. Transient Transformation of D. longan EC

The DNAMAN 9.0 software was used to design specific amplification primers at the 3′ and 5′ ends of the CDS sequence of Dlo013167 (DlbZIP60), with the upstream and downstream primers containing desired cleavage sites at the 5′ end, and cloned into the pCAMBIA1301-35S-GUS vector (Table S3). The pCAMBIA1301-35S-DlbZIP60:GUS construct was transformed into *Agrobacterium tumefaciens* (EHA105). The bacteriophage containing recombinant plasmid was activated and cells were collected by centrifugation at 7800 r/min for 10 min. The cell pellet was resuspended with MS permeabilised containing 3% sucrose, 50 µmol·L^−1^ acetosyringone (AS) and µmol·L^−1^ MgCl_2_. The cells were resuspended and adjusted to OD_600_ between 0.6 to 0.9. *D.* longan EC that had been cultivated for approximately 15 d were transferred to the prepared infiltration solution and co-cultivated for 30 min at 200 r/min. Subsequently, the co-cultured *D.* longan EC were filtered dry. And they were transferred to MS solid medium containing 20 g/L to be cultured for 3 days. Finally, *D.* longan EC in MS medium were subjected to GUS staining to confirm whether the transient transformation were successful or not, and the EC with successful transient transformation were collected in liquid nitrogen freezing and then put into −80 °C refrigerator for storage. 

### 4.10. Measurement of Endogenous Hormones in D. longan EC after Transient Transformation

The physiological indicator kits for the determination of endogenous IAA, ABA and MeJA were purchased from Shanghai Yuanjv Biotechnology Centre (Shanghai, China), and the specific operation procedures were referred to the company’s manual. 

## 5. Conclusions

**Figure 9:**
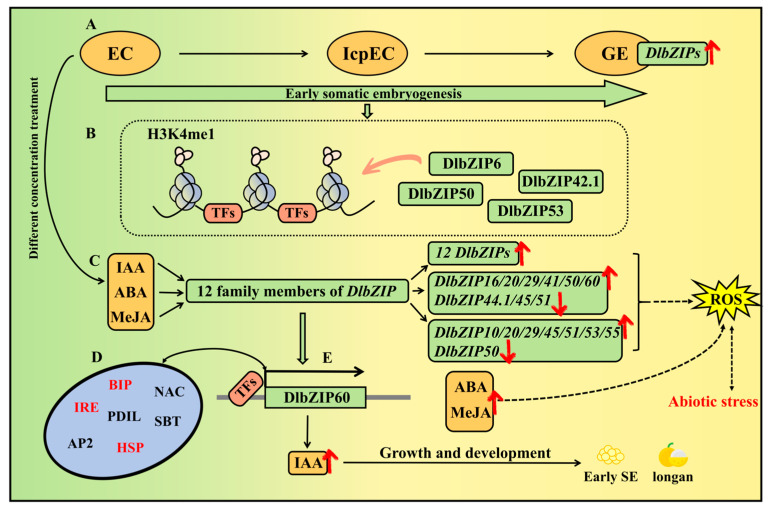
The molecular mechanism and regulatory network of DlbZIPs during early *D.* longan SE. (**A**) *DlbZIPs* were highly expressed in GE stage. (**B**) a total of four members have different H3K4me1 binding peaks in early SE and differentially expressed with increased chromatin accessibility. (**C**) The expression of 12 *DlbZIP* family members were up-regulated or down-regulated under different concentrations of IAA, ABA and MeJA. (**D**) There was a close interprotein interaction with the heat stress proteins HSP70-17 and HSP90-7, and there was an interprotein interprotein interaction with key plant transcription factors such as NAC and AP2. (**E**) *DlbZIP60* could participate in various hormone pathways and affect the development during early SE in *D.* longan. And the ROS system may be affected by changing the content of ABA and MeJA in response to abiotic stress.

To elucidate the role of bZIP TFs in *D.* longan SE, we performed genome-wide identification and expression analysis of bZIP TFs by using the third-generation genome of ‘Honghezi (HHZ)’ *D.* longan, and screened DlbZIP60 for subcellular localization and transient transformation. In this study, we screened and identified a total of 51 members of the DlbZIP family in *D.* longan, which were found to be more closely related to dicotyledonous plants by phylogenetic tree and synteny analysis. The results of *cis*-acting element prediction indicated that *DlbZIP* famliy members may participate in multiple stress responses and hormone regulation. Transcriptome and qRT-PCR analyses revealed that most of *DlbZIP* family members were highly expressed in GE during early SE and promotes the process of *D.* longan SE. There were 51 *DlbZIP* family members with different degrees of response in the transcriptomes of nine different developmental organs of *D.* longan, among which *DlbZIP10*/*20*/*45*/*60* showed a high level of expression in each developmental organ. Most of DlbZIP family members were expressed higher in the 2,4-D hormone-treated group than in the control group (MS) at the same time, presumably they may respond to auxin treatment treatment. The qRT-PCR analysis of *D.* longan SE under different hormone treatments showed that 12 *DlbZIP* family members were up-regulated under IAA treatment. *DlbZIP16*/*20*/*29*/*41*/*50*/*60* were up-regulated under different ABA concentrations. Most of *DlbZIP* family members were up-regulated by 100 μM and 200 μM MeJA, and the highest expression was found in 100 μM MeJA. Combined with the results of above experiments, we selected DlbZIP60 for protein interactions network analysis, and found that DlbZIP60 interacted closely with heat stress proteins HSP70-17, HSP90-7, their molecular chaperones BIP1, BIP2, BIP3, interacted with NAC, AP2 and other plant growth related TFs. The results of subcellular localization and transient transformation showed that DlbZIP60 was located in the nucleus. The changes in the content of endogenous hormones IAA, ABA and MeJA were positively correlated with the expression of *DlbZIP60*. The results showed that *DlbZIP60* has an important role during early SE in *D.* longan and mediated hormones. These findings provide a model in which bZIP TFs respond to early *D.* longan SE and related hormone synthesis pathways (Figure 9).

## Data Availability

All analyzed data for this study are included in the contents of this article and Supplementary Materials.

## Supplementary Materials

The following supporting information can be downloaded at: https://www.mdpi.com/article/10.3390/plants13050662/s1, Figure S1: Alluvial diagram of *DlbZIP* genes divided into three types (type I to II and other). Type I to II stand for differentially accessible *DlbZIP* genes between RNA-seq, ATAC-seq and H3K4me1 database. Other stand for no difference in three databases. The right is the representative gene of each type. Table S1. Analysis of physicochemical properties of DlbZIP family members. Table S2. The qRT- PCR primer sequences. Table S3. Vector construction primer sequences.
