# Supplementary material for: Genome-Wide Identification and Expression Analysis Reveal bZIP Transcription Factors Mediated Hormones That Functions during Early Somatic Embryogenesis in Dimocarpus longan
Source: Plants (Basel). 2024 Feb 28;13(5):662. doi: 10.3390/plants13050662 (PMC10934284; doi:10.3390/plants13050662)
Supplement: Supplementary file 1 [file plants-13-00662-s001.zip › Fig. S1.pdf]

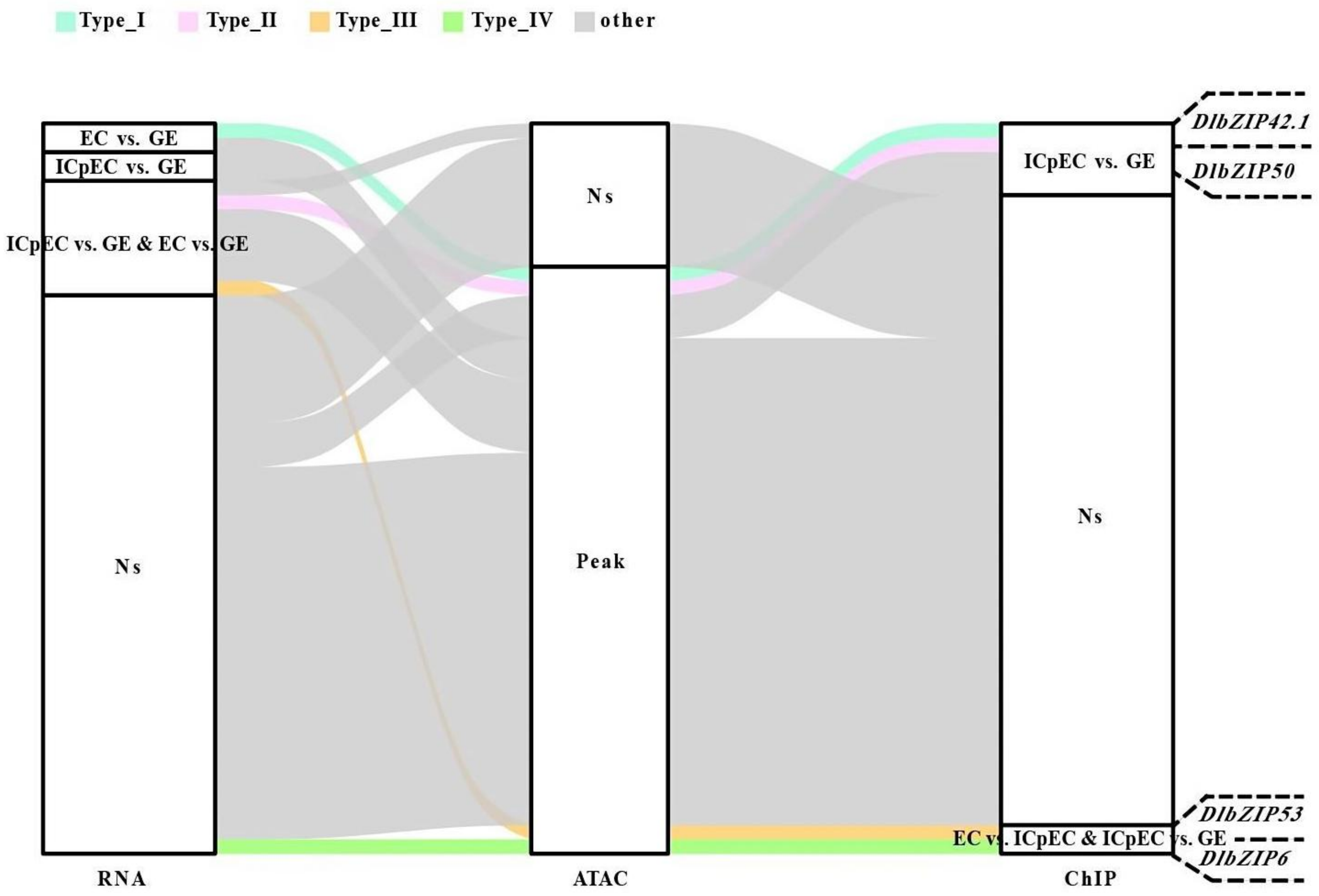

Fig. S1 Alluvial diagram of *DlbZIP* genes divided into three types (type I to II and other). Type I to II stand for differentially accessible *DlbZIP* genes between RNA-seq, ATAC-seq and H3K4me1 database. Other stand for no difference in three databases. The right is the representative gene of each type.
